# Activation of kappa opioid receptors reduces EEG seizure activity in a mouse model of temporal lobe epilepsy

**DOI:** 10.1186/1471-2210-11-S2-A10

**Published:** 2011-09-05

**Authors:** Luca Zangrandi, Christoph Schwarzer

**Affiliations:** 1 Department of Pharmacology, Innsbruck Medical University, 6020 Innsbruck, Austria

## Background

Neuropsychiatric disorders are one of the main challenges of human medicine with epilepsy as one of the most common and serious disorders of the brain. Temporal lobe epilepsy represents the most common type of epilepsies and is often accompanied by marked neuronal degeneration. One main factor that causes neural loss is the excitotoxicity of glutamate, which is copiously released during seizures and hypoxia accompanying seizures. There is evidence that endogenous opioids, namely dynorphin (Dyn), act as modulators of neuronal excitability. It was also shown that the deletion of proDyn in mice and low expression in humans is associated with increased epilepsy vulnerability. Dyn targets opioid receptors and in particular the κ opioid receptor (KOP). The KOP receptors in the hippocampal formation are located in very strategically points for the control of the glutamate release and most important they are not altered under epileptic conditions. Interestingly, proDyn expression is reduced after an initial increase in most epilepsy models and activation of KOP receptors may be neuroprotective. Still, the functional background of these neuroprotective effects is not fully understood. The aim of this study was to investigate the influence of KOP agonists and antagonists on EEG patterns of epileptic mice.

## Methods

Kainic acid (KA; 3 nmol in 50 nL saline) was injected unilaterally into the dorsal hippocampus, causing acute and delayed behavioral and EEG effects. Four-channel EEG traces were recorded from ipsi-and controlateral hippocampi and motorcortices applying depth and surface electrodes, respectively. The KOP-specific agonist U-50,488H and antagonist GNTI were dissolved in saline (adjusted to pH 7.4) and applied i.p. or intracisternally, respectively.

## Results

Sharp waves and paroxysmal discharges in the ipsilateral hippocampus were recorded about 2 weeks after KA injection. Paroxysmal discharges were accompanied by behavioral arrest and stereotypic behaviour like head nodding. Application of KOP agonists at different doses (2, 5, 10 mg/kg) markedly reduced paroxysmal discharges. In contrast, the KOP antagonist (3 nmol) prolonged the duration of such discharges. These data represent observations from preliminary experiments.

## Conclusion

Data collected so far confirm the anticonvulsant action of KOP agonists in the subchronical phase of epilepsy, suggesting that neuroprotective effects are indeed due to reduced seizure activity.

